# Importance of physician history taking in complementing patient-reported interstitial lung disease questionnaire

**DOI:** 10.1186/s12890-022-02294-3

**Published:** 2022-12-27

**Authors:** Tal Moshe Perluk, Inbal Friedman Regev, Ophir Freund, Eyal Kleinhendler, Tzlil Hershko, Sharona Ben-Ami, Amir Bar-Shai, Avraham Unterman

**Affiliations:** 1 Institute of Pulmonary Medicine, Tel Aviv Medical Center, Tel Aviv University, Tel Aviv, Israel; 2 Department of Internal Medicine B, Tel Aviv Medical Center, Tel Aviv University, Tel Aviv, Israel; 3 Center of Excellence for Interstitial Lung Diseases, Institute of Pulmonary Medicine, Tel Aviv Medical Center, Tel Aviv University, Weizmann Street 6, 6423906 Tel Aviv, Israel

**Keywords:** Diagnostics, Interstitial lung disease, Chest questionnaire, Pulmonary fibrosis

## Abstract

**Background:**

Patient-reported interstitial lung disease (ILD) questionnaires are commonly used for the evaluation of ILD patients. However, research to test their performance is scarce.

**Methods:**

This study aimed to assess the performance of the Chest Questionnaire in consecutive ILD patients presenting to a tertiary ILD center. The results of Chest Questionnaires routinely filled by patients were analyzed together with clinical and demographic data retrieved from the patients’ medical records. The ability of each questionnaire item to detect positive findings, such as environmental and occupational exposures, was examined relative to any additional findings detected by physician-acquired history. History was obtained by an experienced ILD pulmonologist who had access to the results of the questionnaire during the clinic visit.

**Results:**

The final cohort for analysis included 62 patients. Shortness of breath frequency and duration were the questionnaire items with the lowest probability of being filled out by patients. The questionnaire performed well in identifying 96.2% of patients with a positive family history and 90.9% of patients with occupational exposures. However, exposures to mold or birds were frequently missed, self-reported by only 53.1% of exposed patients. Questionnaire’s performance was also lower for other exposures associated with ILD (48.3%). An ILD-related exposure was less likely to be identified by the questionnaire in males (*p* = 0.03), while age had no such effect.

**Conclusions:**

The Chest Questionnaire performed well in several domains, while failing to detect some relevant exposures. Therefore, its use should be accompanied by careful history taking by the physician.

## Background

Interstitial lung diseases (ILD) are a heterogeneous group of rare disorders diffusely involving the lung’s parenchyma, of both known and unknown etiologies. The term ILDs encompasses over 200 different pulmonary disorders with various causes and outcomes [1]. An accurate diagnosis of the specific ILD is important for treatment and prognosis but is often challenging [2]. A detailed assessment of medical history is an important part of the diagnostic process and may uncover relevant exposures to inhaled substances that are associated with specific ILDs [1, 3, 4]. In the case of chronic hypersensitivity pneumonitis (HP) identifying an inciting antigen was shown to be associated with improved survival [3]. Because of the vast diversity of exposures relevant to ILD, it is hard to comprehensively assess all during a patient’s visit to the physician’s office. To that end, several patient questionnaires were developed and are commonly used to assist in identifying the cause of ILD [5], and their use is suggested by international guidelines [6]. However, there are no validated or internationally accepted questionnaires, and research to test their performance in clinical practice is scarce [5].

The Chest Interstitial and Diffuse Lung Disease Patient Questionnaire (hereafter, the Chest Questionnaire) is a widely used questionnaire developed in partnership with the American College of Chest Physicians [7]. This study aims to evaluate its performance in the assessment of ILD patients, in the settings of an expert ILD center.

## Methods

### Settings

This retrospective study assessed the performance of the Chest Questionnaire in consecutive ILD patients presenting to a tertiary ILD center of excellence at the Tel Aviv Medical Center in Israel. All patients first presenting to our ILD clinic are routinely asked to fill out the Chest Questionnaire, to aid the pulmonologist in diagnosing the etiology of ILD. The Questionnaire was handed out by a clinical coordinator, which was available for questions as needed. The only instructions given were to fill-out all items of the questionnaire prior to entering the physician’s office. The results of these prospectively filled questionnaires were analyzed together with retrospective clinical and demographic data retrieved from the patients’ medical records. This study was approved by the local ethics committee (approval number 0287-21) and was conducted in accordance with the declaration of Helsinki.

### Study population

The study population included consecutive patients presenting for the first time to our expert ILD clinic during the first half of 2021. We included patients with ILD of any cause, providing that they completed at least one item from the questionnaire. Exclusion criteria were age under 18 years and failure to fill out at least one questionnaire item.

### Data extraction

We used a formal Hebrew translation of the Chest Questionnaire [7], which was forward and back-translated to assure accuracy. This Hebrew version of the questionnaire is routinely used in several ILD clinics in Israel. The following data points were extracted from the Chest Questionnaire: symptoms, smoking history, family history, exposures, occupational history, chronic co-morbidities, and medication history.

For each patient, we obtained a detailed demographic and medical history from the medical records. Clinical data were acquired only from the clinic visit that took place after the patient filled-out the questionnaire. Clinical data included the ILD diagnosis (obtained through a multidisciplinary discussion according to recent guidelines [1, 4]), relevant family history, and any relevant prior exposures. The pulmonologist had access to the results of the questionnaire during the clinic visit. Therefore, only new findings identified in the clinic visit are regarded as being identified by the pulmonologist.

### Data analysis and definitions

To assess the performance of the questionnaire, we compared the findings in each item with any additional findings detected by an experienced ILD physician (A.U, the head of our ILD center of excellence) during the office visit. Performance was assessed for each questionnaire item and full performance was defined as the ability to identify all positive findings using only the questionnaire, without any new findings identified by the physician. To analyze patients’ compliance with filling out each item of the questionnaire, we calculated the number of incomplete or missing answers for each item. For analysis purposes, multiple exposures in the same item of the questionnaire were counted as one. In addition, if there was at least one new finding detected by the physician during the clinic visit for any specific item, it was considered with the group of “additional findings by physician”. We analyzed the ILD-related exposures from the questionnaire that were identified for each patient. ILD-related exposures were defined as any exposure with a known association to ILD or that could influence the patient respiratory symptoms. An ILD specialist reviewed all the exposures in the questionnaire to decide on their relevance to ILD based on previous literature [1, 4]. The only exposure category from the questionnaire that was not considered to be necessarily related to ILD is “animals” (item 12), which is a general term that was only deemed relevant if the patient wrote a specific animal known to be related to ILD (such as pigeons). All other exposures from the questionnaire (items 12, 15 and 17–19) were included, providing there was a known association to ILD. A diagnosis of post-corona virus disease 2019 (COVID-19) ILD was determined for patients that suffered from moderate-severe COVID-19 disease and had evidence of residual radiologic ILD (fibrotic or non-fibrotic) with relevant symptoms such as dyspnea, cough and functional deficit [8]. In our analysis and throughout the manuscript, males and females are in reference to the patient sex as identified by the physician during the patient encounter. Items 13, 14, and 20 were not included in our analysis because of their open nature.

### Statistical analysis

Baseline patient data are reported as mean with standard deviation or median with interquartile range. Chi-square test and student’s t-test were performed to compare patients with and without an ILD-related exposure, for categorical and continuous variables, respectively. A *p* value under 0.05 was considered statistically significant. Data were analyzed using “R” version 4.0.2 and Microsoft Excel.

## Results

### Baseline characteristics and ILD-related exposures

Sixty-three ILD patients met the inclusion criteria, of which one patient was excluded for not filling out any question in the questionnaire. The final cohort for analysis included 62 patients. Of these, 51.6% were male, the mean age was 67.9 ± 11.8, and 64.5% were past or current smokers. Table 1 describes the baseline characteristics of the cohort population. About a third of patients (34%) suffered from severe or frequent cough. A similar percentage (37%) had shortness of breath during regular walking or milder activity.

**Table 1:** Baseline characteristics of the cohort population

	Total n = 62
Age, mean ± SD, years	67.9 ± 11.8
Male sex	32 (51.6)
Smoking status	
Never	22 (35.4)
Past	32 (51.6)
Current	8 (12.9)
Diabetes	18 (29)
Hypertension	31 (50)
Heart disease	20 (32.3)
Gastroesophageal reflux disease	27 (43.5)
ILD MDD diagnosis	
Idiopathic pulmonary fibrosis	9 (14.5)
Connective tissue disease related-ILD	17 (27.4)
Hypersensitivity pneumonitis	13 (21)
Post COVID-19	7 (11.3)
Others	16 (25.8)
Cough	
None/rarely	16 (25.8)
Occasionally	18 (29)
Most days	12 (19.4)
Often or in severe attacks	9 (14.5)
Shortness of breath	
Only during strenuous exercise	7 (11.3)
Hurrying or walking uphill	18 (29)
Regular walking	16 (25.8)
After 100 yards	3 (4.8)
Minimal activity	4 (6.5)

Numbers represent the number of patients (and % in parentheses), unless stated otherwise. Cough and shortness of breath as reported by the Chest Questionnaire items 1 and 5, respectively. *COVID-19* corona virus disease 2019, *ILD* interstitial lung disease, *MDD* Multidisciplinary discussion

ILD-related exposures were identified in 37 patients (59.7%), either by the questionnaire or during the clinic visit, as presented in Table 2. Patients with Hypersensitivity pneumonitis had the highest rate of ILD-related exposures (85%), while none of the post-COVID-19 patients had a known exposure (Table 2). Of note, seven out of nine patients with idiopathic pulmonary fibrosis (IPF) had an ILD-related exposure, five to an HP-related exposure and two to asbestos. However, these exposures were not considered as relevant after careful multidisciplinary discussion. Age or sex were not found to be associated with the presence of ILD-related exposures.

**Table 2:** Analysis of selected variables by ILD-related exposures

	Total n = 62 (%)	No exposure^a^ n=25 (%)	Positive exposure^a^ n=37 (%)	*p*^b^
Age, mean ± SD, year	67.9 ± 11.8	64.9 ± 11.4	69.9 ± 11.7	0.107
Male sex	32 (51.6)	15 (60)	17 (45.9)	0.277
ILD multidisciplinary discussion diagnosis				
IPF	9 (14.5)	2 (8)	7 (18.9)	0.231
CTD-ILD	17 (27.4)	9 (36)	8 (21.6)	0.213
HP	13 (21)	2 (8)	11 (29.7)	0.039
Post COVID-19	7 (11.3)	7 (28)	0	< 0.001
Other	16 (25.8)	5 (20)	11 (29.7)	0.390

*COVID-19* corona virus disease 2019, *CTD* connective tissue disease, *HP* hypersensitivity pneumonitis, *IPF* idiopathic pulmonary fibrosis, *ILD* interstitial lung disease, *MDD* Multidisciplinary discussion

^a^Any occupational or environmental exposure from the questionnaire that is known to be associated with ILD, and was identified during the medical visit, or using the Chest Questionnaire

^b^*p* values were calculated using *T* test and Chi-square test for continuous and categorical variables, respectively

### Questionnaire performance

Positive findings in the different items of the questionnaire and additional findings identified by the physician at the office visit are described in Table 3. Occupational exposures (questionnaire item 17), HP-related exposures (item 12), and other specific exposures (item 19) were detected in 11 (17.7%), 32 (51.6%), and 29 (46.8%) patients, respectively. The Chest Questionnaire had the highest performance for detecting known comorbidities (item 7), family history (item 10), and occupational exposures (item 17), being the source for positive findings in 86.4%, 96.2%, and 90.9%, respectively. Five patients had active cancer that was found only by the physician, and it was the main comorbidity that was not encountered by the questionnaire. ILD-related medical conditions (item 21), such as inflammatory bowel disease or connective tissue disease, were present in 26 patients (41.9%) and discovered by the questionnaire in 81% of them. HP-related exposures (item 12) were missed by the questionnaire and discovered only by the physician in 15 patients (46.9%), all with exposure to mold or birds. The questionnaire performance was also low for other specific exposures, with positive findings in only 48.3% of all patients with any such exposure.

**Table 3:** Positive findings identified by the Chest Questionnaire or by the physician

Questionnaire item	Positive findings in questionnaire^a^ n (%)	Additional findings by physician^a^ n (%)	Total^a^ n
7—Known comorbidities	38 (86.4)	6 (13.6)	44
8—Recreational drug use	2 (50)	2 (50)	4
10—Family history	25 (96.2)	1 (3.8)	26
12—HP related exposures			
Mold	8 (57.1)	6 (42.9)	14
Birds	8 (52.1)	11 (57.9)	19
Others	15 (100)	0	15
Any HP exposure	17 (53.1)	15 (46.9)	32
17—Occupational exposures	10 (90.9)	1 (9.1)	11
18—Work location	0	0	0
19—Other specific exposures			
Animals	8 (44.4)	10 (55.6)	18
Metals	4 (57.1)	3 (42.9)	7
Food/plant	5 (100)	0	5
Miscellaneous	5 (62.5)	3 (37.5)	8
Skilled	6 (85.7)	1 (14.3)	7
Any general exposure	14 (48.3)	15 (51.7)	29
21—ILD related medical problems	21 (80.8)	5 (19.2)	26

*HP* hypersensitivity pneumonitis, *ILD* interstitial lung disease

^a^Multiple exposures per patient are counted as one

In a subgroup analysis including only patients with an ILD-related exposure, such exposure was less likely to be identified by the Chest Questionnaire among males than females (29% vs. 65%, *p* = 0.03). Age was similar between patients with identified and missed ILD-related exposure (68.6 ± 12 vs. 71.2 ± 11, *p* = 0.51).

### Patient compliance

Patient compliance in filling out the questionnaire items is described in Table 4. Shortness of breath frequency and duration were the items with the lowest compliance, with 22.6% and 62.5% missing answers, respectively. High rates of missing answers were also found for cough duration (20.5%) and occupational history (16.1%). In addition, smoking duration was filled only partially by 16 patients (25.8%).

**Table 4:** Patient compliance with the questionnaire items*

Item	Full answer, n (%)	Partial answer, n (%)	Missing, n (%)
1—Cough frequency	56 (90.3)	0	6 (9.7)
2—Cough duration	31 (79.5)	0	8 (20.5)
3—Cough at night	37 (94.9)	0	2 (5.1)
4—Productive cough	35 (89.7)	0	4 (10.3)
5—SOB frequency	48 (77.4)	0	14 (22.6)
6—SOB duration	9 (37.5)	0	15 (62.5)
7—Known comorbidities	59 (95.2)	3 (4.8)	0
7a—CTD-suggestive symptoms	56 (90.3)	5 (8.1)	1 (1.6)
8—Recreational drug use	60 (96.8)	0	2 (3.2)
9—Smoking duration	45 (72.6)	16 (25.8)	1 (1.6)
10—Family history	55 (88.7)	4 (6.5)	3 (4.8)
11—Old house residency	60 (96.8)	0	2 (3.2)
12—HP related exposures	57 (91.9)	4 (6.5)	1 (1.6)
15—Dust or smoke exposure	55 (88.7)	0	7 (11.3)
16—Occupational history	52 (83.9)	0	10 (16.1)
17—Occupational exposures	60 (96.8)	0	2 (3.2)
18—Work location	59 (95.2)	0	3 (4.8)
19—General exposures	60 (96.8)	0	2 (3.2)
21—ILD related medical problems	60 (96.8)	0	2 (3.2)

*HP* hypersensitivity pneumonitis, *SOB* shortness of breath, *CTD* connective tissue disease

* Because of their open nature, items 13, 14, and 20 were not included in our analysis

## Discussion

In this study, 62 patients were screened for possible occupational and environmental exposures and other factors related to ILD, using both the Chest Questionnaire and history acquired by the pulmonologist during the office visit. To the best of our knowledge, this is the first attempt to test the performance of the different parts of this commonly used questionnaire.

We found that the Chest Questionnaire performed well in identifying occupational exposures (91%) but performed to a lesser extent in detecting HP-related exposures (53%) or other specific exposures (48%). This may allude to potential weaknesses in the design of this questionnaire leading to decreased sensitivity in some important domains, compared to direct history-taking by a qualified physician. It also strengthens the need for a skilled history taking to complement this- and probably any- questionnaire. Mold, birds, and other animal exposures were frequently recognized only by the physician. Potential explanations may include patients’ lack of understanding of the terms or comprehension of environmental hazards or even deliberate misleading. A striking example was from a patient with chronic HP who did not report having 100 pet canaries in the questionnaire, fearing the physician’s response. This was uncovered by the physician during the medical encounter, leading to antigen avoidance.

Interestingly, male patients provided less diagnostic exposure information in the questionnaires compared to females. This phenomenon of male under-reporting was previously demonstrated in several studies [9].

As expected, relevant exposures were common among patients with hypersensitivity pneumonitis (p = 0.04), an exposure-related disease, and not common in patients with COVID-19 related ILD (p < 0.001), a condition unrelated to occupational or environmental exposures. Less obvious is the fact that most IPF patients and nearly half of connective tissue disease-related ILD (CTD-ILD) patients had an occupational or environmental exposure. A recent meta-analysis concluded that exposure to metal dust, wood dust, pesticides, and occupational history of farming or agriculture increased the risk for IPF [10]. It is plausible that the risk conferred by such exposures is not limited to IPF but extends to other types of ILDs, including CTD-ILD. In assessing patients’ compliance with filling out the questionnaire, certain domains were more frequently missed. Most patients (62.5%) who reported shortness of breath (SOB), did not respond when asked about its duration. SOB frequency and cough duration were also neglected frequently (22.6%, 20.5% respectively). A possible explanation might be the insidious nature of SOB in patients with ILD.

Although most patients successfully reported all their comorbidities (86%) and ILD-related medical problems (81%), a missed comorbidity can be valuable for diagnosis and treatment. Failure to fully report one’s comorbidities can be explained by forgetting one of the medical conditions among highly comorbid patients or simply by misunderstanding their importance. A history of previous or active cancer (5 patients), which is not even included in the Chest questionnaire, may also be of relevance. A positive family history was identified by the questionnaire in almost all cases (96%), while the use of recreational drugs in only 50%. Patients might be unwilling to reveal all their personal data in the setting of a questionnaire compared to their personal meeting with the physician.

While some questionnaire items may have lower performance than others, its overall clinical importance should not be underestimated. By relying solely on the questionnaire, occupational exposures and HP-related exposures would have been discovered in 90.9% and 53.1%, respectively. This information could be highly valuable, especially to general healthcare practitioners less experienced in evaluating ILD patients [3, 4]. Clinicians can use this exposure information to provide counseling for the patient and prevent further harm [11, 12]. During a short patient encounter, only a small amount of time is spent on most topics [13]; by reviewing the questionnaire, the physician can quickly identify information important for the diagnosis or management of ILD patients.

The study’s limitations are its relatively small sample size and its reliance on questionnaires that are subjective and are affected by patients’ compliance and recall biases. While the questionnaires were prospectively filled out by consecutive patients, data extraction from medical records and data analysis were retrospective. Another limitation is the lack of gold standard for exposure history, which means that other ILD-related exposures might be missed by both the physician and the questionnaire. This fact highlights the limited detection rate of some questionnaire items, which would have been even lower if more exposures were discovered by the physician. Additionally, the clinician may have been directed in their history taking after reviewing the questionnaire to delve further into exposures not noted by the patient, which emphasizes the conclusion that any questionnaire should always be accompanied by skilled history taking. The study was held at an expert ILD center, which means that a referral bias could not be excluded, and it may also explain the high percentage of HP patients in our study. Finally, the current study design assessed the questionnaire’s performance in identifying ILD-related factors (including exposures, risk factors, co-morbidities, etc.) rather than whether it was helpful in making the diagnosis (for example in the setting of a multi-disciplinary discussion).

## Conclusion

In conclusion, the Chest Questionnaire provides useful information that may aid the clinician in establishing the correct ILD diagnosis and detecting potentially relevant exposures, but cannot replace careful history taking by the physician. We found that the Chest Questionnaire performed well in identifying occupational exposures (91%) or a positive family history (96%). However, its performance was lower in detecting HP-related exposures (53%) or other specific exposures (48%). Hence, attempts should be made to improve these specific domains in this questionnaire. Future studies should assess the clinical utility of a modified questionnaire on larger prospective multi-center cohorts with diverse demographic characteristics. Until this can be accomplished, meticulous face-to-face history-taking by an expert pulmonologist remains crucial to the accurate diagnosis of patients with ILD.

## Data Availability

The datasets used and/or analyzed during the current study are available from the corresponding author on reasonable request.

## Abbreviations

COVID-19: Coronavirus disease 2019
CTD: Connective tissue disease
HP: Hypersensitivity pneumonitis
ILD: Interstitial lung disease
IPF: Idiopathic pulmonary fibrosis
SOB: Shortness of breath
